# What is the best Rocker Shoe design?

**DOI:** 10.1186/1757-1146-5-S1-O6

**Published:** 2012-04-10

**Authors:** Jonathan Chapman, Stephen Preece, Christopher Nester, Bjoern Braunstein, Angela Höhne, Gert-Peter Brüggermann

**Affiliations:** 1 School of Health, Sport and Rehabilitation Sciences, University of Salford, UK; 2 Institute of Biomechanics and Orthopaedics, German Sport University, Cologne, Germany

## Background

**Figure 1:**
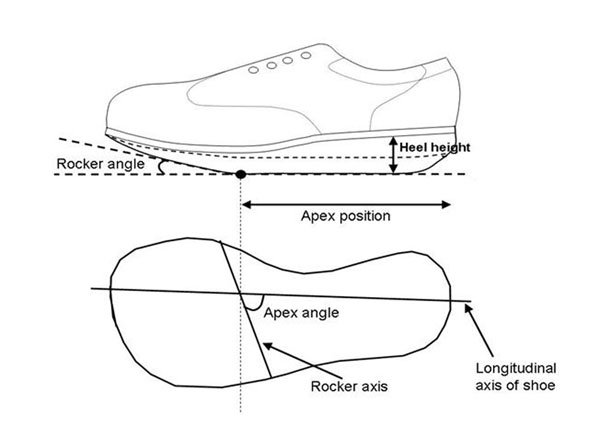
Apex position, rocker angle and apex angle in a rocker shoe

Rocker shoes are often prescribed to reduce in-shoe pressures in order to minimise the risk of ulceration in diabetic patients. However, the efficacy of the 3 principal design features of a rocker shoe (apex position, rocker angle and apex angle, see Figure 1) is unknown. Only one known study to date has systematically varied 2 of the 3 design features [1]. Therefore the aim of this study was to investigate the effect of the three principal design features, quantify inter subject variability and establish whether there is any difference in the response of the diabetic and the healthy cohort by recording in shoe plantar pressure.

## Materials and methods

By using 12 different rocker shoe designs and a control shoe, we systematically varied each design feature apex position (50-70% of shoe length), rocker angle (10-30°) and apex angle (70-100° to longitudinal shoe axis). For each shoe, peak 1st metatarsophalangeal joint (MPJ) pressure was measured during walking. Data was collected from 30 diabetic and 30 healthy subjects and repeated measures ANOVA used to investigate the mean effect of each feature. Descriptive statistics were used to investigate inter-subject variability and a two-way ANOVA was used to compare the response between the diabetic and healthy cohort.

## Results

All three design features had a significant effect on peak 1st MPJ pressure. However, there was considerable inter-subject variability in the optimal rocker angle and optimal apex position. In contrast, an apex angle of between 90-100° resulted in minimal pressures across almost all subjects.

## Conclusion

The results suggest that pressure offloading can be achieved by employing an apex angle of approximately 95°. However, rocker angle and apex position should be chosen on individual by individual basis.
